# Endometrial Cancer Stem Cells: Where Do We Stand and Where Should We Go?

**DOI:** 10.3390/ijms23063412

**Published:** 2022-03-21

**Authors:** Constanze Banz-Jansen, Laureen P. Helweg, Barbara Kaltschmidt

**Affiliations:** 1 Department of Gynecology and Obstetrics, and Perinatal Center, Protestant Hospital of Bethel Foundation, University Medical School OWL at Bielefeld, Bielefeld University, Campus Bielefeld-Bethel, Burgsteig 13, 33617 Bielefeld, Germany; constanze.banz-jansen@evkb.de; 2 Forschungsverbund BioMedizin Bielefeld, OWL (FBMB e.V.), Maraweg 21, 33617 Bielefeld, Germany; barbara.kaltschmidt@uni-bielefeld.de; 3 Department of Cell Biology, University of Bielefeld, Universitätsstrasse 25, 33615 Bielefeld, Germany; 4 Molecular Neurobiology, Bielefeld University, Universitätsstrasse 25, 33615 Bielefeld, Germany

**Keywords:** endometrial cancer, cancer stem cells, endometrial cancer stem cells, MYC, NF-κB, PD-1, PD-L1, mitochondria

## Abstract

Endometrial cancer is one of the most common malignant diseases in women worldwide, with an incidence of 5.9%. Thus, it is the most frequent cancer of the female genital tract, with more than 34,000 women dying, in Europe and North America alone. Endometrial Cancer Stem Cells (CSC) might be drivers of carcinogenesis as well as metastatic and recurrent disease. Therefore, targeting CSCs is of high interest to improve prognosis of patients suffering of advanced or recurrent endometrial cancer. This review describes the current evidence of molecular mechanisms in endometrial CSCs with special emphasis on MYC and NF-κB signaling as well as mitochondrial metabolism. Furthermore, the current status of immunotherapy targeting PD-1 and PD-L1 in endometrial cancer cells and CSCs is elucidated. The outlined findings encourage novel therapies that target signaling pathways in endometrial CSCs as well as immunotherapy as a promising therapeutic approach in the treatment of endometrial cancer to impede cancer progression and prevent recurrence.

## 1. Endometrial Cancer

Endometrial cancer (EC) is one of the most common malignant diseases in women worldwide. Annually, about 320,000 women are diagnosed with EC. In high-income countries especially, the incidence of EC is elevated at 5.9% [1]. In Europe and in North America, it is the most frequent cancer of the female genital tract. Major risk factors are obesity, diabetes type II, physical inactivity and elevated estrogen levels in postmenopausal women [2,3]. Romania in particular shows high incidence and mortality rates with frequent comorbidities for endometrial cancer such as hypertension, obesity and HPV infection [4,5]. In most cases, EC is presenting early on with postmenopausal bleeding and therefore often is diagnosed at an early stage. However, more than 34,000 women die of EC in Europe and North America each year [6], and incidence is rising. The prognosis for recurrent EC in general is miserable, with a five-year overall survival from 15% to 17% [7]. Endometrial cancers have been classified in two categories. Type 1 includes grade 1 and 2 endometrioid carcinomas, which are linked to chronic estrogen stimulation and arise from complex atypical hyperplasia. They often show good prognosis, since they are diagnosed early. Type 2 comprises grade 3 endometrioid and non-endometrioid cancers that develop from atrophic endometrium with a poorer prognosis [6]. Recently, the cancer genome atlas research project revealed a genomic re-classification of EC with four distinct subtypes: DNA polymerase epsilon catalytic subunit (POLE) ultramutated, microsatellite instability (MSI), copy-number low and copy-number high [8].

In clinical treatment, standard therapy is surgical intervention, where surgical staging is state of the art. The surgical procedure for locally restricted endometrial cancer is a total hysterectomy and bilateral adnexal removal with or without pelvic and paraaortic lymphadenectomy laparoscopically [9]. For localized recurrences, surgery or radiation or a combination of both may be an option. Nonlocalized recurrent tumors are usually treated with systemic therapy. The options are hormonal treatment, chemotherapy and/or rather novel alternatives like targeted immunotherapy. To understand how EC metastasizes could help to prevent recurrence or at least to improve therapy. According to early research by Virchow, chronic inflammation might be a major driver of tumor development [10]. Furthermore, it was shown that tumors can develop by single cell somatic mutations. This mutated cell was supposed to proliferate locally and when reaching a certain number starts to metastasize [11]. Some tumors develop drug resistance quite rapidly and show clonal heterogeneity, which may be caused by a small subpopulation of cells that show stem-like features [12,13]. The concept of cancer stem cells (CSCs) is the most promising concept until now to fill the gap in understanding cancer progression and resistance.

CSCs are of enormous clinical interest since this small subpopulation of cancer cells may be responsible for tumor growth, invasion and metastasis as well as treatment resistance and cancer relapse. Due to their role in treatment failures, cellular models of CSCs are highly promising tools for the investigation of the underlying molecular biology and the development of novel cancer therapeutics. The purpose of this review is to characterize the role of endometrial cancer stem cells, to describe the molecular pathways involved in ECSC sustainment and the impact of interfering with these pathways as well as to give an outlook of potential CSC-targeting therapeutic implications for endometrial cancer.

## 2. Literature Data Searching

For this narrative review, we performed research on pubmed.gov, clinicaltrials.gov and google.scholar using the keywords ‘endometrial cancer’, ‘endometrial cancer stem cell’, ‘endometrial neoplasm’, ‘endometrioid carcinoma’, ‘uterine cancer’ and ‘uterine cancer stem cell’ together with specific keywords like ‘tumorigenicity’, ‘signaling’, ‘stemness’, ‘pluripotency’, ‘immunotherapy’ and ‘metabolism’ as well as ‘wnt’, ‘PI3K’, ‘Akt’, ‘notch’, ‘CD133/C44‘, ‘nfkb’, ‘nuclear factor kappa b’, ‘RelA’, ‘myc’, ‘mitochondria’, ‘PD-L1’ and ‘pembrolizumab’. Medical subject heading terms used were ‘endometrial neoplasm’, ‘uterine neoplasm’, ‘endometrioid carcinoma’. We sorted the results by most recent and included all papers from 2010 to 2022. For further information, we also included older publications.

## 3. Endometrial Cancer Stem Cells

Most cancers arise in tissues containing a subpopulation of stem cells, which are responsible for the development and maintenance of the respective tissue. The presence of CSCs has been assumed for several decades, as many tumors exhibit significant heterogeneity regarding their morphology, genetic lesions, cell proliferation kinetics and response to therapy even when the tumor initiated from a single cell [14]. The first possible relationship between cancer and stem cells was found in the hematopoietic system, when Fialkow and colleagues showed stem cell involvement in chronic myelocytic leukemia and acute non-lymphocytic leukemia [15,16]. Hierarchical tumor organization was also shown in solid cancers, as for instance only CD44+/CD24-/low tumorigenic breast cancer cells were able to form tumors that contained additional CD44+/CD24-/low cells, as well as phenotypically different non-tumorigenic cells [17]. Consistently, EC shows a hierarchical organization that contains a small subpopulation of tumor-initiating cells able to initiate tumors with morphologies similar to the parental one [18]. The properties of these relatively rare tumor-initiating subpopulations are strongly related to those of normal stem cells, as they are able to proliferate, self-renew and differentiate into diverse cell types of the respective tumor population. In contrast to normal stem cells, CSCs have a specific molecular signature and deregulations of several molecular signaling pathways [19].

The discovery of (E)CSCs drastically changed the perspective of cancer research regarding tumor biology and approaches of treatment. They are most commonly identified using specific cell-surface antigens like CD44, CD133 and CD24 together with implantation and tumor sphere formation assays to prove their ability to initiate a tumor containing the same CSCs as well as differentiated progeny [20]. Next to tumor sphere formation assays, invasion, migration and chemoresistance assays are conducted to confirm the tumorigenic phenotype of ECSCs [21,22]. Consistently, subpopulations isolated from endometrial cancer cell lines showed stem cell properties such as self-renewal, low proliferative activity, chemoresistance and tumor initiation [23]. CD133/CD44^+^ endometrial cancer cells were able to form tumor spheres, showed enhanced chemoresistance and were able to initiate tumor formation with the same phenotype as the parental tumor when transplanted into immunodeficient mice [24]. Further, CD133/CD44^+^ cells isolated from endometrial cancers expressed the pluripotency markers Myc, Sox-2, Nanog and Oct4 as well as other stemness-related genes such as Nestin and showed enhanced clonogenic ability and sphere formation [25]. As CSCs rely on self-renewal, it is most likely that they originate from normal stem cells and utilize the already implemented self-renewal pathways. However, it has been shown that CSCs can also be derived from progenitor cells suggesting that oncogenic mutations include the regain of self-renew ability [26]. This stands in line with the fact that the origin of CSCs has been connected to dedifferentiation of a mature cancer cell with epithelial to mesenchymal transition (EMT) [27]. Consistently, upregulation of EMT-associated genes like TWIST1 and SNAI1 was demonstrated in an endometrial cancer stem-like cell line and treatment with EMT-blocker salinomycin inhibited the tumorigenicity of these cells [21]. An aldehyde dehydrogenase (ALDH)-1^high^ cancer stem-like cell subpopulation isolated from other endometrial cancer cell lines also showed increased expression of EMT-associated genes like SNAI1 as well as pluripotency markers Sox2 and Nanog [28]. Furthermore, ALDH-1^high^ ECSC spheres show expression of Oct4, Nanog and Myc and inhibition of ALDH activity suppressed tumor sphere formation and decreased their chemoresistance [29]. The significantly increased expression of ALDH1 and epithelial cell adhesion molecule (EpCAM) as well as Oct4, Nanog, Sox2 and Myc was also revealed in a CD133^+^ cell subpopulation isolated from an endometrioid adenocarcinoma [30]. Hypoxia was further shown to promote an endometrial cancer stem-like cell phenotype by increasing the expression of CD133, ALDH1, Oct4, Sox2 and Nanog and enhancing tumor sphere formation [31]. Signaling pathways involving miRNAs were demonstrated to be involved in modulating endometrial CSC properties [32]. For instance, miR-423 and miR-135a contribute to CSC characteristics as they promote proliferation, migration, invasion and chemoresistance of endometrial cancer cells [33,34]. Furthermore, miR-101 has been shown to suppress EMT, self-renewal and invasiveness of aggressive endometrial cancer cells by decreasing the expression of TWIST1, ALDH1 and Nanog [35]. However, other signaling pathways like Wnt/β-Catenin, Notch and phosphatidylinositol 3-kinase (PI3K)/AKT were also demonstrated to regulate stemness in endometrial cancer [21,32,36].

## 4. Targeting Signaling Pathways in Endometrial Cancer Stem Cells

There are several pathways that have been described to maintain stemness and mediate resistance in endometrial CSCs. The Wnt/β-Catenin pathway is one of these and also has been described to control self renewal and promote growth and migration in a variety of CSCs [37]. In endometrial cancer cells, activation of Wnt/β-Catenin signaling was shown to facilitate tumor progression and accelerate cell growth as well as promote tumor migration and invasion [38,39,40]. Further, miR-15a-5p-mediated inhibition of Wnt/β-Catenin signaling suppressed cell proliferation and stemness of endometrial cancer cells, as it regulates the expression of various stemness genes like Oct-4, Sox2 and Nanog [41]. Additionally, SMOC-2-mediated activation of Wnt/β-Catenin enhanced chemoresistance of CD133/CD44+ endometrial CSCs [22]. Another pathway known to modulate stemness and resistance of CSCs is the Notch signaling pathway [42]. A study around Mitsuhashi revealed that expression of Notch receptors and ligands was higher in endometrial cancer than the normal endometrium and is associated with higher grade and myometrial invasion [43]. However, another study suggested that the Notch pathway may act as an endometrial cancer suppressor, as expression of Notch molecules was reduced compared to the adjacent non-carcinogenic tissue [44] Nonetheless, treatment of endometrial cancer cells with Notch inhibitor DAPT suppressed their invasiveness [43]. Furthermore, expression of Notch was significantly higher in CD133^+^ cells than CD133^-^ cells and inhibition of Notch increased cell cycle arrest and apoptosis [45]. In primary endometrial CSCs, miR-134 overexpression led to the downregulation of Notch pathway proteins as well as suppressed proliferation, migration and drug resistance [36]. A study investigating estrogen activated Notch signaling in ER-positive and -negative endometrial cancer cell lines showed a proliferative effect in both cell lines, although only ER-positive cells showed activated Notch signaling and reduced cell viability upon inhibition of Notch [46]. In ER-negative cells, estrogen mediated proliferation was induced by PI3K/AKT signaling, which is another master regulator of CSCs [47]. Several studies revealed the importance of PI3K/AKT signaling in endometrial cancer cells, as it promotes cell proliferation, migration as well as mediates chemoresistance [48,49]. For instance, human epidermal growth receptor 2 (HER2) mediated PI3K/AKT activation increased paclitaxel resistance in endometrial cancer cells [50]. Consistently, inhibition of PI3K/AKT signaling by, for instance, metformin suppressed viability, sphere formation, migration and invasion as well as induced apoptosis, which are features contributing to the CSC phenotype [51,52]. Further, dysregulations of several miRNAs affect PI3K/AKT signaling and contribute to the invasive, EMT and CSC phenotype of tumor cells including endometrial cancer [32].

### 4.1. Targeting NF-κB in Endometrial Cancer Stem Cells

The nuclear factor ‘kappa light chain enhancer’ of activated B-cells (NF-κB) belongs to a family of transcription factors involved in important cellular processes, but also plays a major role in the progression of numerous diseases including cancer [53]. Furthermore, NF-κB-mediated signaling pathways promote tumor progression by directly being involved in maintaining stem cell characteristics of CSCs [54]. Regarding endometrial cancer, all NF-κB members and related proteins were found frequently expressed [55]. Disruption of NF-κB signaling in endometrial cancer cells has been shown to induce G1 cell cycle arrest through the transcriptional down-regulation of Cyclin-dependent kinase 4 (CDK4) expression [56]. Furthermore, non-canonical NF-κB member RelB signaling was found to be elevated in endometrioid adenocarcinomas and connected to tumor initiation and tumor growth in vivo [57]. A molecular profiling study of CD44 and ALDH expressing endometrial tumor circulating cells found increased expression of NF-κB member RelA associated with tumor infiltration and EMT [58]. Consistently, miRNA-16-mediated suppression of RelA activation inhibited the invasion and migration of endometrial stromal cells often involved in endometrial pathogenesis [59]. Invasion and migration of endometrial cancer cells was also inhibited via IL-37b-mediated suppression of RelA, however it did not affect EMT [60]. Furthermore, analysis of the cancer genome atlas RNAseq data revealed a correlation between transcriptional activation of NF-κB p65-regulated genes with FXYD5/dysadherin mRNA levels in endometrial cancer, which is stronger expressed in higher staged or invasive ECs [61]. NF-κB has been shown to promote endometrial cancer cell survival under hypoxia [62], which is known to modulate chemo- and radioresistance, as well as having been suggested to constitute niches for CSCs [63]. This stands in line with post-radiation endometrial cancer recurrences showing increased nuclear translocation of NF-κB members p50, RelB and cRel [64]. Regarding endometrial CSCs, nanopore sequencing of several CSC populations isolated from different tissues including endometrial cancers showed highly enriched genes involved in the GO-term “NF-κB binding” [19]. We recently published a significant survival decreasing effect of the NF-κB Inhibitor dexamethasone and pyrrolidine dithiocarbamate (PDTC) in CSCs derived from non-small cell lung cancer, which may also affect endometrial CSCs with elevated NF-κB activity [65].

NF-κB signaling is further directly connected with signaling pathways known to maintain stemness of endometrial CSCs. For instance, AKT activates NF-κB through stimulation of the trans-activating domain and activation of the inhibitor of kappa B (IκB) kinases (IKK) [66]. Consequently, AKT-mediated NF-κB activation in endometrial cancer has been shown to increase Cyclooxygenase-2 (COX-2) expression as well as estradiol-mediated vascular endothelial growth factor (VEGF) and basic fibroblast growth factor (bFGF) [67,68]. NF-κB signaling is further directly connected to Notch signaling, as several cross-regulations have been observed [69]. Some cross-talks can also be found between Wnt/β-Catenin and NF-κB signaling, such as a Wnt5a/NF-κB feedback loop that sustains both elevated Wnt5a levels and NF-κB activity [70].

### 4.2. Targeting Myc in Endometrial Cancer Stem Cells

The Myc family of transcription factors are one of the few master regulators of oncogenesis, as deregulation has been detected in over 70% of human cancers and associated with poor prognosis [71]. A study conducting immune histochemical stainings found a 78.3% positive rate of c-Myc in endometrial cancer tissues and an amplified c-Myc in 25% of the cases [72,73]. Additionally, high expression of c-Myc was more often observed in low differentiated endometrial cancers than moderately differentiated ones [73]. Regarding endometrial carcinogenesis, upregulation of c-Myc in endometrial cancer cells was shown to induce EMT, drug resistance and invasion [74,75]. Consistently, targeting c-Myc using small molecule JQ1 inhibited endometrial cancer growth in cell culture and xenograft models [76].

The emerging role of Myc in CSCs is becoming increasingly clear, as several studies demonstrated a central role of c-Myc in maintaining stem-like properties in a variety of cancers including breast and colon cancer, and inhibition of Myc suppressed their stemness [77,78]. Increased expression of c-Myc also was observed in several stem-like endometrial cancer subpopulations [25,30], making c-Myc an important target to eliminate endometrial CSCs. We recently published a significant survival decreasing effect utilizing the small molecule KJ-Pyr-9 in CSCs derived from colon and lung cancer [65,79]. In ovarian cancer, overexpression of the tumor suppressor miRNA-654 reduced cell proliferation and induced cell death by acting against Myc-, Akt- and Wnt-signaling pathways, which are also important in the regulation of endometrial CSCs [80]. In endometrial cancer cells, 17β-estradiol and tamoxifen were shown to induce c-Myc expression through ER-α36-mediated activation of ERK and AKT, which in turn promoted cell proliferation. Additionally, inhibition of PI3K significantly reduced cell growth [81]. Suppression of tumor driver α-Enolase in endometrial cancer cells significantly reduced proliferation and invasion in vitro, as well as tumorigenicity in vivo, by the inactivation of PI3K/AKT signaling. This inactivation led to reduced c-Myc expression as well as Snail and N-cadherin and the overexpression of PI3K in α-Enolase silenced endometrial cancer cells reversed this effect [82]. However, c-Myc promoter binding protein 1 (MBP-1), an alternative translation product of α-Enolase, has been shown to suppress c-Myc expression, which reduced the proliferation and migration of endometrial cells [83]. Targeting the Hedgehog signaling pathway, which has been suggested to maintain CSCs [84], significantly inhibited the growth of endometrial cancer cells with a concomitant reduced expression of cyclin D1 and N-Myc [85]. Furthermore, nanopore sequencing of CSCs derived from three endometrial tumors further revealed an upregulation of Myc as well as genes associated with the mitochondrion, which stands in line with the fact that amplified Myc expression has also been connected to increased mitochondrial oxidative phosphorylation [19,86].

**Figure 1:**
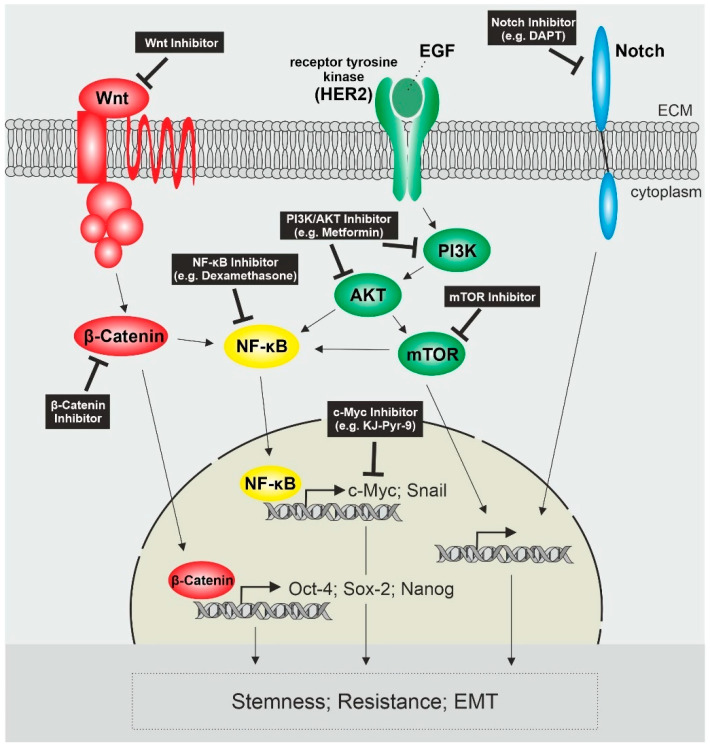
Activated Signaling Pathways in Endometrial Cancer Stem Cells and possible Target Sites. Activated Wnt/β-Catenin Signaling leads to the expression of stemness genes Oct-4, Sox-2 and Nanog as well as activates NF-κB. NF-κB is also activated by the PI3K/AKT/mTOR pathway, whose activation is mediated by tyrosine receptor kinases like HER2 and leads to the expression of genes like c-Myc and Snail. Another receptor contributing to cancer stem cell characteristics is Notch. Possible target sites affecting these pathways as well as stemness, resistance and invasiveness of CSCs are pictured in the black boxes. PI3K = phosphatidylinositol 3-kinase; HER2 = human epidermal growth factor receptor 2; EGF = epidermal growth factor; ECM = extracellular matrix; mTOR = mammalian target of rapamycin.

Taken together, the most prominent activated pathways in endometrial cancer stem cells include Wnt/β-Catenin, PI3K/AKT signaling and Notch signaling in crosstalk with other signaling pathways like NF-κB, which leads to the upregulation of genes associated with stemness, resistance and EMT (Figure 1).

## 5. Mitochondrial Metabolism in Endometrial Cancer Stem Cells

In contrast to the differentiated tumor bulk surrounding CSCs that shows persistent activation of aerobic glycolysis, many CSCs show metabolic plasticity and are able to switch their metabolic state to favor glycolysis or oxidative metabolism [87]. For instance, pancreatic CSCs prefer oxidative metabolism, as they show an increased mitochondrial mass as well as decreased lactate production [88]. Consistently, nanopore sequencing of CSCs isolated from various tissues demonstrated an upregulation of mitochondrial and oxidative phosphorylation pathways as well as glycolytic pathways further underlining the metabolic plasticity of CSCs [19]. On one hand, endometrial CSCs displayed higher mitochondrial membrane potential, reactive oxygen species, ATP levels, and oxygen consumption rates than regular endometrial cancer cells and knock-down of mitochondrial peroxiredoxin three decreased sphere formation and reduced their cellular viability [89]. Additionally, endometrial CSC spheres showed an increased uptake in glucose associated with lower lactate production and mitochondrial oxidative phosphorylation and endometrial CSCs isolated from endometrial cancer cell lines showed increased mitochondrial mass [28,90]. On the other hand, spheroid endometrial CSCs with ALDH activity show glycolytic dependency and glycolytic suppression impaired their stemness [29]. In gastric CSCs, the glycolytic α-Enolase has been shown to regulate stem cell-like properties by stimulating glycolysis [91]. In endometrial cancer cells, expression of α-Enolase is elevated and correlates with worse outcomes, as well as having been shown to regulate glycolysis, cell proliferation, migration and invasion via the PI3K/AKT pathway [82].

However, independent of their metabolic state, CSCs rely on mitochondrial function as it is crucial for processes like regulation of stemness and chemoresistance [92]. Thus, targeting mitochondrial metabolism may provide a new therapeutical tool for targeting (endometrial) CSC-mediated carcinogenesis. The already clinically approved standard type II diabetes medication Metformin has shown promising results in cancer treatment through the inhibition of oxidative phosphorylation in mitochondria and activation of 5’ AMP-activated protein kinase (AMPK), leading to reduced cell growth and proliferation [93]. However, next to mitochondrial metabolism, Metformin was shown to target CSCs by interfering with pathways like Wnt/β-catenin, TGF-β and NF-κB signaling [94]. Regarding endometrial cancer, a meta-analysis revealed that metformin does not reduce the risk, although it was significantly associated with improved overall survival and reduced recurrence [95]. Metformin treatment of endometrial cancer cells inhibited cell growth via the induction of cell cycle arrest, apoptosis and autophagy as well as decreased EMT [96,97]. Furthermore, metformin significantly reduced estrogen-mediated proliferation and c-Myc expression of endometrial cancer cells [98]. However, high glucose and hypoxia in endometrial cancer negatively affect metformin response in vitro and in vivo [99]. Regarding endometrial CSCs, metformin reduced the number and activity as well as the expression of CSC genes in endometrial CSCs isolated from established endometrial cancer cell lines [28]. Another mitochondria targeting agent is salinomycin [100], which also has been shown to suppress proliferation, migration, sphere formation as well as tumorigenicity and induces apoptosis of CSCs [101] including endometrial CSCs [21].

## 6. Targeted Immunotherapy

Immunotherapy is an emerging innovative cancer treatment that modulates the body’s own tumor immune response. However, due to its complexity, side effects and uncertainty, immunotherapy is still under intensive studies [102]. Immunotherapies can be categorized in two ways: active, where an immune response is actively triggered by transferred cells or vaccines, and passive with transferred antibodies such as immune checkpoint inhibitors. Regarding endometrial cancer, peptide, dendritic cell and nucleic acid based vaccines are currently being trialed amongst others, yet still need to be clinically approved [103]. Passive immunotherapies include immune checkpoint blockage by targeting programmed death ligand 1 (PD-L1) or cytotoxic T-lymphocyte-associated protein 4 (CTL4) as well as bispecific T-cell engager (BiTE) antibodies [104]. As already stated, targeting intracellular signaling pathways that regulate cellular growth and proliferation like Myc and NF-κB represents a promising therapeutic strategy. Other signaling pathways targeted in endometrial cancer include pathways like the mammalian target of rapamycin (mTOR), epidermal growth factor (EGF) and insulin-like growth factor (IGF), where receptors or kinases are targeted by specific inhibitors [105]. For instance, a phase II study using Temsirolimus, a mTOR inhibitor, showed 14% and 4% partial response as well as 69% and 48% stable disease in chemotherapy-naive and -treated patients, respectively [106]. A phase II study in recurrent or metastatic endometrial cancer utilizing the selective inhibitor of the EGF receptor (EGFR) tyrosine kinase activity erlotinib showed a low response rate of 12.5%, although it is well tolerated [107]. However, taking EGFR expression levels/mutations into consideration could improve clinical outcomes, as in vitro and in vivo studies showed an anti-tumor effect of erlotinib only in EGFR high level, although not low level endometrial cancer cells [108].

As the presence of tumor-infiltrating lymphocytes is a favorable prognostic factor in endometrial cancer and indicates an active role of the immune system, targeted therapies like PD-1/PD-L1 checkpoint inhibitors may have the potential to be effective in endometrial cancer [109]. PD-1 and PD-L1 are frequently expressed in endometrial cancer [110], thus evading immune surveillance and response. A meta-analysis conducted for endometrial cancer revealed that PD-L1 expression is not associated with overall survival, yet positively correlated with poor differentiation and advanced tumor stage [111]. Based on this frequent expression of PD-L1, suppression of PD-L1 in endometrial cancer cell lines significantly inhibited cell growth [112]. For instance, metformin treatment of endometrial cancer cell lines heavily decreased the expression level of PD-L1 protein as well as activated co-cultured T cells and thus suppressed cancer cell growth [113]. A study around Hsu and a coworker revealed that PD-L1 expression in CSCs is enriched through an EMT/β-catenin/PD-L1 axis and suppression of this pathway led to the downregulation of PD-L1 and enhanced the amount of tumor-infiltrating activated CD8+ T cells, as well as the efficacy of Tim-3 blockade therapy [114]. In breast cancer, PD-L1+ cells showed higher stemness, in vitro and in vivo, mediated by Notch, and/or the PI3K/AKT pathway as well as PD-L1 was shown to maintain stemness by promoting Oct4 and Nanog expression [115,116]. A recent study further revealed that PD-L1 and PD-L2 expression in ECSCs is increased upon hypoxia and knockdown of PD-L1 resulted in a reduced expression of pluripotency genes and a number of spheres, as well as impaired cell proliferation [117]. Thus, targeting not only cancer cells, but also CSCs through PD-L1 inhibition depicts an important therapeutic strategy to improve clinical outcomes.

Currently, several immune checkpoint inhibitors either targeting PD-1 or PD-L1 and thus interaction with PD-1 and B7.1 are situated in phase II/III trials [118]. In ovarian cancer, chemotherapy induces local immune suppression through NF-κB–mediated PD-L1 up-regulation indicating that a combination of chemotherapy and immunotherapy may improve the antitumor response [119]. Regarding endometrial cancer, dostarlimab, an antibody targeting PD-1 is now in a phase III trial in combination with carboplatin and paclitaxel chemotherapy [120]. Pembrolizumab, an antibody targeting PD-L1 together with lenvatinib demonstrated promising antitumor activity in patients with advanced endometrial cancer, who have experienced disease progression subsequent to systemic therapy [121]. A phase III trial revealed that prembolizumab plus lenvatinib treatment has an overall favorable benefit/risk profile compared to chemotherapy and thus represents a new standard therapy for advanced pre-treated endometrial cancer [122]. However, a Markov model study revealed that pembrolizumab, plus lenvatinib as first-line treatment in advanced high microsatellite stable endometrial cancer, would decrease efficacy and worsen quality of life as well as increase costs in comparison to chemotherapy. For microsatellite instable endometrial cancer, the model predicted that first-line pembrolizumab plus lenvatinib treatment would result in fewer deaths compared to chemotherapy, thus may provide a clinical benefit even though it is not a cost-effective treatment option [123]. Currently, an active phase III trial is comparing the efficacy of pembrolizumab plus lenvatinib to chemotherapy in female participants with Stage III, IV, or recurrent endometrial cancer [124].

Overall, targeting PD-1/PD-L1 in endometrial cancer seems to be a promising therapeutic strategy to not only target cancer cells, but also CSCs with considerable anti-tumor effects to improve clinical outcomes and prevent cancer recurrence.

## 7. Clinical Outlook

Oncological treatment in general is becoming more and more individualized. The main requirement therefore is perfect collaboration between physicians, molecular pathology and geneticists in a molecular tumor board (MTB). The first studies show better progression-free (PFS) and overall survival (OS) for patients who received MTB-based therapy [125]. In the case of endometrial cancer testing for MSI-/MMR status will be mandatory. Another promising tool for screening for metastasis or even early detection will be Liquid Biopsy using blood samples and uterine aspirates (UAs) [126]. While these personalized yet very expensive methods of diagnostics are promising tools in a wealthy healthcare system they will not be affordable for the predominant number of patients in the world. The same will unfortunately be true for personalized therapy with antiangiogenetic and immunotherapy.

## 8. Strength and Limitations of This Review

**Figure 2:**
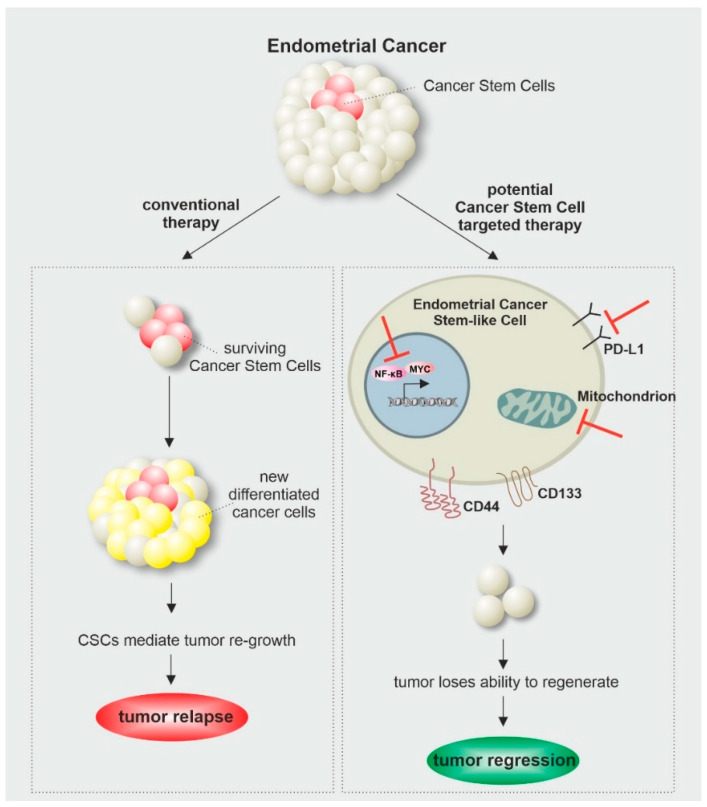
Endometrial Cancer Stem Cell as a therapeutical Target for Cancer Therapy.

As cancer is one of the leading causes of death worldwide, frequent research is needed to expand our knowledge about underlying mechanisms and improving current treatment options. The discovery of CSCs and their contribution to tumorigenesis as well as treatment resistance and cancer relapse changed the perspective of cancer research, as novel therapeutic strategies are needed to overcome CSC-mediated treatment failures. This makes in vitro models highly promising tools to study underlying mechanisms, sustaining the stemness of endometrial cancer cells. As described in this review, interfering with signaling pathways like Myc and NF-κB as well as cellular metabolism showed promising effects in the diminishment of endometrial cancer stem cells and could therefore display considerable therapeutic targets for CSC-directed cancer therapy (Figure 2).

Since endometrial CSCs are a relatively recent discovery, there are only limited studies investigating signaling pathways involved in maintaining endometrial CSC properties. Furthermore, future studies are needed to understand the underlying mechanisms when interfering with these signaling pathways for the development of CSC-targeted therapies.

## 9. Conclusions

Unequivocally, CSCs are highly relevant for the formation, resistance and recurrence of EC. Thus, selectively targeting CSCs is an emerging promising therapeutic strategy in the treatment of EC. Several signaling pathways including Myc and NF-κB are involved in the maintenance and survival of ECSCs and targeting these reduced the invasion and migration ability as well as tumorigenicity. Furthermore, ECSCs show metabolic plasticity, which may contribute to their maintenance as targeting glycolysis, in addition to mitochondrial metabolism, decreased their stemness. The inhibition of immune checkpoints is another clinically relevant ECSCs-targeting strategy, since PD-L1 expression has been shown to maintain stemness of CSCs and targeting PD-L1 in EC has shown promising anti-tumor effects. However, there are only limited data describing the molecular mechanisms that modulate ECSC characteristics making further in vitro and in vivo studies necessary to provide new insights and possibly identify novel molecular targets.
